# The Protein-Independent Role of Phosphate in the Progression of Chronic Kidney Disease

**DOI:** 10.3390/toxins13070503

**Published:** 2021-07-19

**Authors:** Irene Faria Duayer, Eduardo Jorge Duque, Clarice Kazue Fujihara, Ivone Braga de Oliveira, Luciene Machado dos Reis, Flavia Gomes Machado, Fabiana Giorgetti Graciolli, Vanda Jorgetti, Roberto Zatz, Rosilene Motta Elias, Rosa Maria Affonso Moysés

**Affiliations:** 1 LIM 16, Nephrology Department, Hospital das Clínicas HCFMUSP, Universidade de São Paulo, São Paulo 05403-000, Brazil; ireneduayer@gmail.com (I.F.D.); eduardojorgeduque@gmail.com (E.J.D.); clarice.fujihara@fm.usp.br (C.K.F.); ivoneol@usp.br (I.B.d.O.); lucienem@usp.br (L.M.d.R.); flaviamachado@usp.br (F.G.M.); fabianag@usp.br (F.G.G.); vandajor@usp.br (V.J.); roberto.zatz@gmail.com (R.Z.); rosilenemotta@hotmail.com (R.M.E.); 2 Post-Graduation, Universidade Nove de Julho (UNINOVE), São Paulo 01525-000, Brazil

**Keywords:** phosphate, diet, CKD progression, renal fibrosis, autophagy, apoptosis

## Abstract

Several factors contribute to renal-function decline in CKD patients, and the role of phosphate content in the diet is still a matter of debate. This study aims to analyze the mechanism by which phosphate, independent of protein, is associated with the progression of CKD. Adult Munich-Wistar rats were submitted to 5/6 nephrectomy (Nx), fed with a low-protein diet, and divided into two groups. Only phosphate content (low phosphate, LoP, 0.2%; high phosphate, HiP, 0.95%) differentiated diets. After sixty days, biochemical parameters and kidney histology were analyzed. The HiP group presented worse renal function, with higher levels of PTH, FGF-23, and fractional excretion of phosphate. In the histological analysis of the kidney tissue, they also showed a higher percentage of interstitial fibrosis, expression of α-actin, PCNA, and renal infiltration by macrophages. The LoP group presented higher expression of beclin-1 in renal tubule cells, a marker of autophagic flux, when compared to the HiP group. Our findings highlight the action of phosphate in the induction of kidney interstitial inflammation and fibrosis, contributing to the progression of renal disease. A possible effect of phosphate on the dysregulation of the renal cell autophagy mechanism needs further investigation with clinical studies.

## 1. Introduction

Chronic kidney disease (CKD) is considered a public health problem. Its prevalence increases with age and varies between 11 and 13% worldwide [1]. Although CKD is generally an irreversible condition, several approaches can be used to slow down the disease progression, such as control of blood pressure, dyslipidemia and hyperglycemia, use of angiotensin-converting enzyme inhibitor, and a low-protein diet [2,3,4,5,6,7]. Among these strategies, a low-protein diet is the highest debatable proposal for delaying kidney-function decline, due to concerns over its compliance, safety, and efficacy. A lack of consensus on the benefits of reducing protein in the diet relies on the overlapping role of protein and phosphate content in the diet. As protein sources are rich in phosphate, a protein intake reduction is recommended to achieve a restricted phosphate intake. It is possible to restrict protein without restricting phosphate, although the opposite is difficult.

In experimental studies, the effect of a low-protein diet protected CKD models from glomerular damage and proteinuria [8], promoting greater preservation of the glomerular filtration rate [9]. It should be noted that dietary protein and phosphate are highly correlated. Therefore, although some experimental studies have focused on evaluating the isolated effect of protein restriction, they may also have been biased due to inadvertent phosphate restriction. On the other hand, the individual contribution of a phosphate-rich diet to CKD development has been clearly demonstrated [10,11]. A high-phosphate diet was shown to induce more interstitial kidney fibrosis and tubular atrophy, with minimal glomerulosclerosis, in uremic rats. Interestingly, the damage was blunted after phosphate restriction [12]. Of note, the protein content in the diet was not mentioned in this study.

In the clinical scenario, a low-protein diet reduced the concentration of uremic toxins and preserved residual renal function, even in the transition from conservative care to incremental dialysis regimens [13]. Dietary protein restriction also has delayed CKD progression in a recent meta-analysis [14]. However, as protein restriction was correlated with lower serum phosphate levels, this beneficial effect of diet could have been explained in part by the phosphate restriction. The largest prospective study so far, the MDRD [15], showed only a small benefit of a low-protein diet in patients with moderate renal insufficiency, whereas the progression of renal disease was not affected in patients with severe renal insufficiency. Nevertheless, the content of phosphate in the diet was not addressed in this trial and the impact of its individual contribution in slowing CKD progression remains unknown.

It is known that phosphate can lower renal and circulating Klotho [16], an antiaging protein involved in cytoprotection and antifibrosis, with antioxidative and antiapoptotic effects [17]. In addition, defective autophagy in the renal cells due to Klotho deficiency has already been demonstrated [18]. Autophagy is a catabolic process with critical functions in maintaining cellular homeostasis and preserving cell viability in stress conditions. Some pieces of evidence suggest a relationship between autophagy and the progression of CKD through different pathways, including TGF-β signaling [19], a potent fibrogenic factor for the development of renal fibrosis. TGF-β affects several biological processes, regulating cell proliferation and differentiation, apoptosis, and acts by binding to specific transmembrane receptors which induce intracellular signaling mediated by SMAD proteins [20]. Apoptosis is a mechanism of programmed cell death which is different from necrosis. Phosphate has been described as a mediator of apoptosis in experimental models of endothelial [21], osteoblastic [22], and peritoneal mesothelial cells [23]. There is evidence of the participation of apoptotic cell death in the progression of kidney injury [24], but the effect of phosphate on renal cell apoptosis has not yet been elucidated.

Since the mechanism by which a high-phosphate diet accelerates CKD progression is not yet completely elucidated, we hypothesized that it might be associated with a dysfunctional autophagic flow. Therefore, in the current study, we evaluated two groups of Munich-Wistar rats according to phosphate content in their diet (low 0.2% versus high 0.95%) and tested the impact of a low-phosphate diet on the kidneys’ histological and immunohistochemistry changes, including an autophagy marker, beclin-1.

## 2. Results

Hemodynamic, morphological, and biochemical parameters obtained from low- (LoP) and high-phosphate diet (HiP) groups are given in Table 1. As expected, the 5/6-nephrectomy induced a reduction in creatinine clearance and an increase in serum urea and creatinine in both groups. Animals from the HiP group presented lower renal function, as evidenced by higher creatinine and lower creatinine clearance; they also exhibited lower hematocrit and lower final weight. The HiP group presented an increase in serum and fractional excretion of phosphate, as well as in parathyroid hormone (PTH) and fibroblast growth factor-23 (FGF-23). Of note, caudal pressure, initial weight, and urinary albumin excretion were similar between groups.

**Figure 1:**
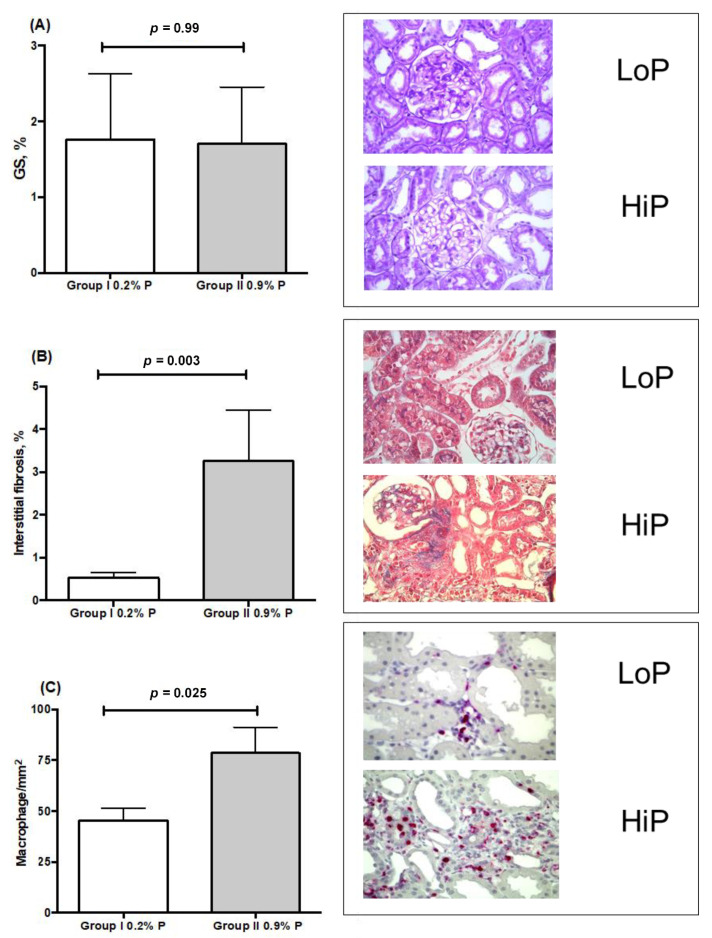
Distribution of histological features between HiP and LoP groups. A low frequency of glomeruli with sclerotic lesions (GS, %) was found 2 months after renal ablation in groups I and II. Periodic acid Schiff (PAS), 200× magnification is shown on the right side (**A**). However, a higher interstitial fibrosis area was found in the HiP group; here demonstrated by graphic and histological representation estimated by Masson (**B**). Additionally, a higher extent of renal infiltration by macrophages (ED-1, cells/mm^2^), was found in the same group (**C**). 200× magnification is shown on the right side. Results expressed as mean ± SD.

**Figure 2:**
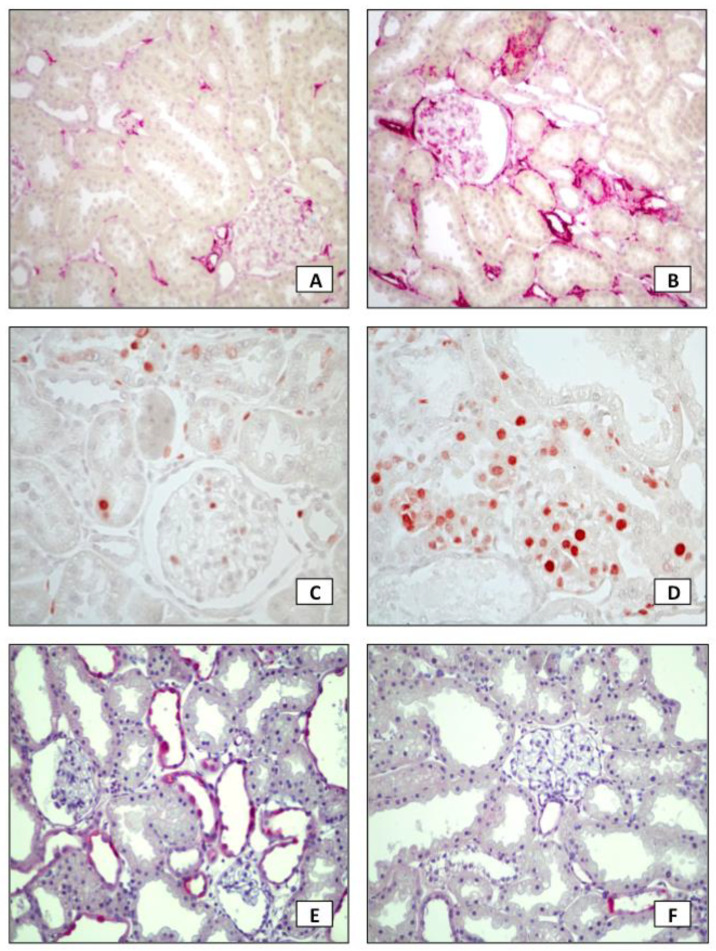
Immunohistochemistry analysis in kidney tissue. α-SMA expression was lower in LoP (**A**) than in HiP (**B**). Likewise, PCNA expression was lower in LoP (**C**) than in HiP (**D**). However, Beclin-1 expression was higher in LoP (**E**) than in HiP (**F**). Positive staining appears as red color; (**A**,**B**,**E**,**F**) magnification ×200; (**C**,**D**) magnification ×400.

**Figure 3:**
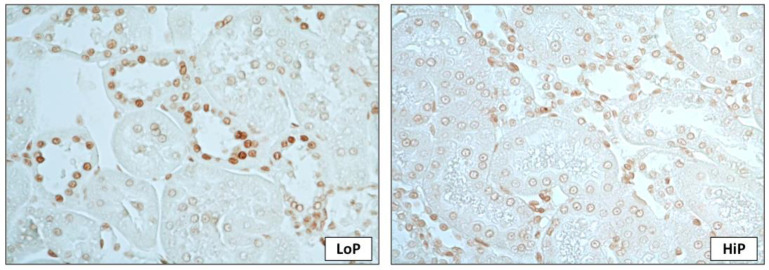
Representative microphotographs of renal interstitial apoptotic cells. More apoptotic cells, analyzed by TUNEL technique, were seen in the LoP group. Magnification of 1.000×.

Histological findings are shown in Figure 1, Figure 2 and Figure 3. Glomerulosclerosis (GS) was barely found in both groups. However, the percentage of interstitial fibrosis was significantly higher among rats in the HiP group. Accordingly, these animals also had a higher percentage of α- smooth muscle actin-stained area and a higher renal infiltration by macrophages, evaluated by ED-1 expression, as shown in Table 2. Phosphorylated SMAD (p-SMAD) expression, a marker of TGF-beta pathway activation, was not different between groups.

Proliferative cell nuclear antigen (PCNA)-positive cells were predominant in the HiP group, suggesting that phosphate overload promoted more intense renal cell proliferation. Conversely, the number of apoptotic cells tended to be higher in the LoP group. Likewise, the beclin 1-positive cells were significantly higher in tubulointerstitial lesions in the LoP group, which indicates a possible effect of higher phosphate in inhibiting the regenerative autophagic process on kidney tissue.

## 3. Discussion

This experimental study investigated whether dietary phosphate restriction is an effective step to slow the progression of CKD, regardless of concomitant dietary protein restriction. We examined the effect of diets with different contents of phosphate (0.2 vs. 0.95%) in a 5/6-nephrectomy animal model, with all animals receiving a low-protein diet (12%). Our findings showed that a high-phosphate diet was associated with hyperphosphatemia, despite the increase in PTH, FGF-23 and, consequently, of the fractional excretion of phosphate. In addition, HiP caused an impairment of renal function and an increase in interstitial fibrosis, which was associated with higher α-actin expression, macrophage infiltration and cellular proliferation, as well as an inhibition of autophagy.

A meta-analysis of 12 cohort studies involving 25,546 CKD non-dialysis patients demonstrated an independent association between serum phosphate levels, kidney failure, and mortality [25]. Moreover, a retrospective cohort study in 13,772 dialysis incident patients has revealed an association between hyperphosphatemia and other abnormalities of CKD-mineral and -bone disorder with a steeper decline of residual kidney function [26]. The mechanism by which phosphate restriction delays CKD progression is yet to be established. In an experimental study, we showed that a phosphate-enriched diet was associated with a more impaired renal function, when compared to a low-phosphate diet in Wistar rats submitted to parathyroidectomy and 5/6-nephrectomy, using a fixed parathyroid hormone (PTH) supplementation [11]. Kusano et al. [10] showed that renal-function protection was associated with a low-phosphate (0.3%) diet, regardless of dietary protein content in uremic rats. Rats with the normal phosphate (0.5%) diets, however, presented these benefits only if fed with a very low-protein (8.4%) diet. Taken together, these results denote that the role of dietary phosphate might be more powerful than dietary protein. However, neither of the above-mentioned studies tried to investigate the potential pathways that could be involved in phosphate-related kidney damage.

The action of phosphate overload in the progression of CKD has been the subject of several studies. Ibels et al. first suggested this possibility in an animal model, but the dietary [27] protein was not controlled, which possibly masked the isolated effect of phosphate. A supposed benefit of a phosphate-independent restriction, rather than protein restriction in the CKD scenario, is of great interest because the risk of controlling serum phosphate together with restricted protein intake can lead to malnutrition and cause higher mortality.

Phosphate diet content does not necessarily determine serum phosphate concentration in CKD patients [28], highlighting the importance of diet restriction independent of serum concentration of this element. The pathological effects of phosphate on bones and the cardiovascular system motivate interventions to treat hyperphosphatemia. In clinical practice, interventions to reduce phosphate levels consist of dietetic restrictions and phosphate binders. Although benefits of this approach on clinical endpoints were questionable in a small-sample-size study [29], in face of the knowledge that phosphate excess is responsible for the overproduction of PTH and FGF-23, it is reasonable to maintain levels of phosphate as low as possible in patients with CKD [30].

A low-protein diet induces low bioavailability of phosphate and decreases serum FGF-23 [31,32], a mediator of sodium-dependent phosphate reabsorption on renal tubular cells. This suggests that the phosphate-regulation mechanism in advanced CKD may somehow be influenced by phosphate intake in an entirely independent way to protein diet content. For this reason, we have evaluated the effect of phosphate diet content on the progression of CKD, in a 5/6-nephrectomy model. A previous experimental study has already demonstrated that phosphate can modify the antiproteinuric response to a very-low-protein diet (0.3 g/kg), showing a correlation between serum phosphate levels and phosphaturia with the antiproteinuric response to a restricted protein diet, independent of the renal function [33]. In a crossover study that included 11 patients with CKD (eGFR 30–55 mL/min/m^2^), the circadian rhythm of serum phosphate was altered with a high-phosphate diet, which was not explained by urine phosphate excretion, PTH, or FGF-23 [34]. This result strengthens the importance of phosphate diet restriction in CKD patients as an independent therapeutic target.

Hemodynamic status does not seem to be involved in the worsening of renal function in the HiP group, as arterial pressure was similar in both groups. Additionally, we did not find significant glomerular damage in our model, which could be explained by the protein restriction. However, we noted more interstitial fibrosis in the HiP diet group.

Interstitial fibrosis is well correlated with loss of renal function in humans and rats. A previous study has associated the development of renal fibrosis induced by a high-phosphate diet via peptidyl-prolyl isomerase Pin 1 [35], whereas other papers have pointed out the harmful role of *α*Klotho deficiency on kidney tissue fibrosis [36,37]. The genetic deletion of Klotho, in experimental models, causes aging, growth retardation, and calcifications [38]. FGF23-Klotho signaling inhibits renal phosphate reabsorption. In the CKD setting, Klotho expression is reduced in the renal tubules [39]. Moreover, the phosphate-rich diet has exacerbated *α*Klotho deficiency in an ischemia-reperfusion acute kidney injury model and further promoted increased kidney tissue fibrosis, accelerating the progression of CKD [18]. The influence of phosphate diet on the renal expression of Klotho was already demonstrated in murine models [40]. Possibly, among other pathways, phosphate overload worsens the renal function by modulating Klotho expression on tubular cells; it should be further investigated in future studies.

Apoptosis and fibrosis are mediated by cytokines, such as TGF-β [41,42]. We have described the association of secondary hyperparathyroidism and consequent higher TGF-β expression on the cardiac remodeling process [43]. Supposedly, there is an association between PTH activation and TGF-β signaling which stimulates the proliferation of fibroblasts, collagen synthesis, and fibrosis. There is even experimental evidence of an integrated action of TGF-β and PTH receptors on bone cells, regulating bone-remodeling signaling [44]. The role of TGF-β/Smad signaling in kidney disease has already been described. TGF-β binds a transmembrane receptor, triggers Smads proteins phosphorylation, and promotes their translocation to the nucleus for transcriptional effects. Therefore, they are essential for the signal transduction of members of the TGF-β family. Indeed, the overexpression of Smad2 and Smad3 proteins in the fibrotic kidney in animal models with CKD has already been demonstrated [45]. Interestingly, there is evidence of Smads activation by other mediators independent of TGF-β, such as advanced glycation end products [46] and angiotensin II [47]. As the effect of hyperphosphatemia on the progression of CKD was not influenced by PTH replacement in an experimental model [11], we wonder whether phosphate would have a direct role on kidney Smad signaling. In our model, there was no influence of hyperphosphatemia on the expression of p-Smad in renal tubular cells. However, this does not rule out the possibility of TGF-β pathway activation independent of Smads phosphorylation. Further studies are needed to investigate this alternative pathway.

In the evaluation of bone tissue, phosphate overload has induced the apoptosis of osteoblast-like cells [22]. Rojas et al. demonstrated in bone biopsies of transplanted patients that the number of apoptotic cells was higher in those with low phosphate and PTH would have an effect in increasing osteoblasts and preventing apoptosis [48]. This inhibitory effect of PTH on bone cell apoptosis has been demonstrated previously [49]. Besides the effects on bone tissue, apoptotic pathways are also related to acute renal failure [48] and could be a possible mediator on the progression of CKD. In our model, we observed a superior proportion of cells undergoing apoptosis in the LoP group, without statistical relevance. These considerations suggest an impaired apoptosis mechanism caused by hyperphosphatemia. However, we must keep in mind that it was not possible to evaluate the exclusive effect of phosphate on kidney tissue apoptosis once animals also have developed PTH and FGF-23 elevation.

In the current study, we addressed another possible mechanism of renal impairment by a high-phosphate diet, the autophagy dysfunction, by measuring the protein beclin-1, a regulatory protein required for autophagosomal membrane nucleation [50]. Autophagy is a catabolic pathway that involves the degradation of cellular components through the lysosomes [51,52]. Beclin-1 phosphorylation has been considered as the autophagy-specific signal for phosphatidylinositol-3-phosphate kinase complex activation and recruitment to the autophagosome biogenesis membrane sites [53]. The knockdown of beclin-1 suppresses autophagy [54] and ischemia-reperfusion increases its levels [55]. The reduction in beclin-1 and the concomitant upregulation of mTOR decrease the growth of malignant cells, highlighting the pro-survival effect of autophagy [52].

The antifibrotic effects of αKlotho were also associated with an upregulated autophagy process, a pathway through which ischemic injury was attenuated in the AKI model and impaired by high-phosphate levels [18]. Experimental studies have shown that autophagic flux mitigates macrophage infiltration and phosphate-induced renal fibrosis. In addition, a phosphate-rich environment induces mitophagy in tubular cells in vitro [56]. Our data showed that the phosphate content in the diet also modulated the expression of beclin-1 in renal tubules in vivo. A HiP diet was also associated with more cellular proliferation and a trend towards a lower apoptotic rate, as depicted by PCNA and TUNEL analysis, respectively. However, in our model, it was not possible to identify whether this occurred as a direct effect of phosphate overload or because of abnormalities in PTH and FGF-23 levels, induced by hyperphosphatemia. This finding needs to be confirmed in a clinical scenario.

A key advantage of this study was to assess the impact of phosphate restriction in a low-protein diet on CKD progression, which included investigation of its influence on histological patterns and molecular phenotypes of kidney tissue. As far as we know, this is the first study to demonstrate the modulation of beclin-1 expression, an autophagy marker on renal tubular cells, by phosphate diet in vivo. The main limitation was the impossibility of disentangling the effect of hyperphosphatemia from other CKD-MBD-related abnormalities, such as the elevation of PTH and FGF-23. We were also unable to evaluate a diet with intermediate phosphate content or renal Klotho expression. However, our model reproduces the real-life condition, where a HiP diet is usually associated with hyperparathyroidism and high levels of FGF-23.

## 4. Conclusions

In summary, this study provided evidence that phosphate diet plays a role in CKD progression, possibly increasing interstitial fibrosis. Furthermore, a phosphate-rich diet might modify the kidney autophagy process. The identification of a protein-independent pathway through which phosphate worsens renal function may open an important avenue for the development of novel approaches aimed at preventing or reducing CKD progression in a clinical setting.

## 5. Materials and Methods

**Figure A1:**
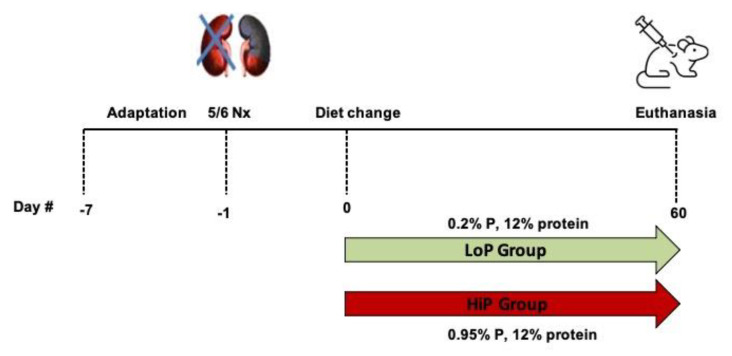
Experimental design of the study. Nx, nephrectomy.

Adult male Munich-Wistar rats obtained from a local facility were included in this study. Rats were maintained at 23 ± 1 °C, with air humidity at 60 ± 5%, under a 12:12-h light–dark cycle. All animals had free access to tap water and were fed a low-protein diet (12% protein). Animals were sacrificed two months later when blood, urine, and renal tissue were analyzed. Tail-cuff pressure, creatinine clearance, and daily urinary albumin excretion were determined in all rats. The experimental design is shown in Appendix A (Figure A1).

All experimental procedures were approved by the local Research Ethics Committee (CEUA #090/10, approved date on 10 August 2010) and developed in strict conformity with our institutional guidelines and with international standards for manipulation and care of laboratory animals. Nephrectomy was performed in one step under anesthesia with pentobarbital sodium, 50 mg/kg ip, by removal of the right kidney and infarction of two-thirds of the left kidney by the closure of two or three branches of the left renal artery. Sham-operated rats underwent anesthesia and manipulation of the renal pedicles without renal mass reduction. After recovering from anesthesia, the animals were returned to their original cages, which were warmed during the following 24 h.

### 5.1. Analytic Techniques

Sixty days after Nx, animals were anesthetized with sodium thiopental (50 mg/Kg BW), and blood samples were collected. Serum creatinine concentration was assessed by a colorimetric method. Serum albumin, phosphate, urea, calcium, and hematocrit were measured by standard methods. PTH was measured by ELISA (Immutopics, San Clemente, CA, USA). FGF-23 was measured using an ELISA kit (KAINOS, Japan) according to the manufactures’ protocol. We calculated glomerular filtration by creatinine clearance based on serum and urine creatinine levels and expressed in ml/min, computed with the formula: urine creatinine (mg/dL) × urine flow (mL/min)/serum creatinine (mg/dL). Urinary albumin was determined by radial immunodiffusion.

Fractional excretion of phosphate (FEP) was obtained through of urine and serum phosphate (mg/dL), urine and serum creatinine (mg/dL), and expressed in %, with the following formula: (urine/serum phosphate)/ (urine/serum creatinine) × 100.

### 5.2. Experimental Groups

Animals were divided into two groups according to phosphate content on diet, as specified below:

Low-Phosphate diet (LoP): *n* = 20; animals received TD-90016, 12% protein diet, modified for a 0.2% phosphate concentration.

High-Phosphate diet (HiP): *n* = 19; animals received TD-90016, 12% protein diet, modified for a 0.95% phosphate concentration.

### 5.3. Histological Techniques

The remnant kidney was perfused in situ at the observed MAP with saline solution and then with Duboscq-Brasil solution for fixation. After being weighed, two midcoronal renal slices were postfixed in buffered 4% formaldehyde. The renal tissue was then embedded in paraffin and prepared for histomorphometry, and immunohistochemistry as described previously [57].

All morphometric evaluations were performed in 4-μm-thick sections by a single observer blinded to the groups. For each rat, the severity of glomerulosclerosis (GS) was estimated, in sections stained with periodic acid Schiff (PAS), by assigning to each glomerulus (at least 120 per rat were examined) a score to estimate the sclerosed fraction of the tuft area, and deriving a GS index (GSI) for each rat as described previously [58]. To assess the degree of interstitial expansion, the percentage of renal cortical area occupied by interstitial fibrosis (%IF) was estimated in Masson-stained sections by a point-counting technique [59].

### 5.4. Immunohistochemistry

Four μm-thick, paraffin-embedded renal sections were mounted on glass slides coated with 6% silane. Sections were deparaffinized, rehydrated, and then heated in citrate buffer to enhance antigen retrieval. They were preincubated with 5% normal rabbit (for ED-1) or horse (for α-actin) serum in Tris-buffered saline (TBS) or casein-based Protein Block solution (Protein Block; DAKO Corporation, Santa Clara, CA, USA (for PCNA, pSmad, and Beclin)), to prevent nonspecific binding. Incubation with the primary antibody was carried out overnight at 4 °C. Negative controls were performed by omitting the primary antibody.

The following primary antibodies were used: a monoclonal mouse anti-rat ED-1 antibody (Serotec, Oxford, UK), for macrophage detection; mouse monoclonal anti-alpha smooth (Sigma-Aldrich, St. Louis, MI, USA), for α-actin detection; polyclonal anti-Beclin 1 antibody (Boster, Pleasanton, CA, USA), for autophagy analysis. After being washed, sections were incubated with biotinylated anti-mouse IgG (H+L), (Vector, Burlingame, CA, USA) for 45 min, followed by VECTASTAIN^®^ ABC-AP Kit, Vector^®^ Red (Vector, Burlingame, CA, USA) for 30 min. Sections were then developed with a fast-red dye solution.

For monoclonal mouse anti-proliferating cell nuclear antigen (Dako, Denmark) and phosphor-smad2 (Ser465/467) antibody (Cell Signaling, Danvers, MA, USA), endogenous peroxidase inhibition was performed with 3% hydrogen peroxide solution and methanol for 30 min. The sections were then incubated overnight with the primary antibody in a humidified chamber at 4 °C. The next day the sections were incubated with biotinylated anti-mouse IgG (H+L) or biotinylated anti-rat IgG (H+L) (Vector, Burlingame, CA, USA) for 45 min, followed by incubation with VECTASTAIN^®^ ABC-HRP Kit (Vector, Burlingame, CA, USA) for 30 min. Sections were then developed using a 3-amino-9-ethylcarbazole substrate chromogen (AEC) (Sigma Chemical, St. Louis, MO, USA). All the sections were rinsed in distilled water, counterstained with Mayer’s Hemalum solution (Merck, Darmstadt, Germany) covered with Kaiser’s glycerin-gelatin (Merck, Darmstadt, Germany).

For interstitial renal-density estimation of ED-1, p-Smad, Beclin, and PCNA, the number of positive cells/mm^2^ was evaluated in a blinded manner at 400× magnification. To evaluate the α-actin we utilized a point count technique, using a microscope with a 100-point ocular connected to a video monitor. We analyzed 25 fields under 3200 magnification. The results were expressed in percentages considering the total area.

### 5.5. Apoptosis

Apoptosis was determined in the renal tubular cells by the TUNEL technique (TdT-mediated X-dUTP Nick end labeling) using the instructions provided by Apoptag plus Peroxidase in Situ Apoptosis Detection Kit (S7101, Millipore Sigma, MA, USA). The apoptotic cells were analyzed in 25 fields, with a magnification of 1000×, to obtain final values expressed in cells/mm^2^.

### 5.6. Statistical Analysis

Differences between LoP and HiP groups were tested using an independent Student’s *t*-test or a Mann-Whitney test accordingly. Relationships between variables were examined by the Spearman correlation coefficient. Data are presented as mean ± SE unless indicated otherwise. A *p*-value of <0.05 was considered significant. Analyses were performed using IBM SPSS Statistics, version 20.0.1 (IBM Corp., Armonk, NY, USA) and GraphPad Prism 9.1.2 statistical software (Graphpad, San Diego, CA, USA).

## Figures and Tables

**Table 1:** Hemodynamic, morphological, and biochemical parameters.

	LoP (0.2% P) *n* = 20	HiP (0.95% P) *n* = 19	*p* Value
Initial weight, g	240 ± 2	241 ± 3	0.746
Final weight, g	315 ± 4	287 ± 8	0.002
Caudal pressure, mmHg	194 ± 4	185 ± 6	0.231
Urinary albumine, mg/24 h	17 ± 4	19 ± 4	0.849
Creatinine clearance, mL/min	0.65 ± 0.04	0.49 ± 0.05	0.019
FE of Phosphate, %	0.7 ± 0.3	84 ± 7.8	<0.0001
Hematocrit, %	44 ± 1	40 ± 1	0.0002
Serum creatinine, mg/dL	0.9 ± 0.03	1.1 ± 0.06	0.026
Serum urea, mg/dL	89 ± 3	83 ± 4	0.302
Serum phosphate, mg/dL	5.8 ± 0.4	9.5 ± 0.7	<0.0001
Ionized calcium, mmol/L	1.24 ± 0.06	1.08 ± 0.05	0.072
FGF-23, pg/mL	306 ± 37	4435 ± 610	<0.0001
PTH, pg/mL	560 ± 178	3644 ± 70	<0.0001

LoP, low-phosphate diet; HiP, high-phosphate diet; FE, fractional excretion; PTH, parathyroid hormone; FGF-23, fibroblast growth fator-23.

**Table 2:** Immunohistochemistry analysis in kidney tissue.

	LoP (0.2% P) *n* = 13	HiP (0.95% P) *n* = 12	*p*
α-SMA, %	0.48 (0.2; 0.8)	2.92 (2; 3.8)	<0.01
ED-1, cell/mm^2^	45 (27; 67)	74 (42; 93)	<0.01
p-SMAD, cell/mm^2^	75.8 (18; 128)	86.7 (19; 149)	0.71
PCNA, cell/mm^2^	67.7 ± 22	167.3 ± 50	<0.01
Apoptosis, cell/mm^2^	267.3 ± 179	173.3 ± 59	0.09
Beclin, cell/mm^2^	10.4 ± 0.7	8.3 ± 0.5	0.024

LoP, low-phosphate diet; HiP, high-phosphate diet; α-SMA, alpha-smooth muscle actin; p-SMAD, phosphorylated SMAD; ED-1, anti-Macrophages antibody; PCNA, proliferative cell nuclear antigen; %, percent of stained area; cells/ mm^2,^, number of positive cells per mm^2^; Data are represented as mean ± SD or as median (IQR).

## Data Availability

The data presented in this study are available on request from the corresponding author.

## References

[1] Hill N.R., Fatoba S.T., Oke J.L., Hirst J.A., O’Callaghan C.A., Lasserson D.S., Hobbs F.D. (2016). Global prevalence of chronic kidney disease—A systematic review and meta-analysis. PLoS ONE.

[2] Wen C.P., Chang C.H., Tsai M.K., Lee J.H., Lu P.J., Tsai S.P., Wen C., Chen C.H., Kao C.W., Tsao C.K. (2017). Diabetes with early kidney involvement may shorten life expectancy by 16 years. Kidney Int..

[3] Kakinuma Y., Kawamura T., Bills T., Yoshioka T., Ichikawa I., Fogo A. (1992). Blood pressure-independent effect of angiotensin inhibition on vascular lesions of chronic renal failure. Kidney Int..

[4] Fried L.F., Orchard T.J., Kasiske B.L. (2001). Effect of lipid reduction on the progression of renal disease: A meta-analysis. Kidney Int..

[5] Ishidoya S., Morrissey J., McCracken R., Reyes A., Klahr S. (1995). Angiotensin II receptor antagonist ameliorates renal tubulointerstitial fibrosis caused by unilateral ureteral obstruction. Kidney Int..

[6] Aldigier J.C., Kanjanbuch T., Ma L.J., Brown N.J., Fogo A.B. (2005). Regression of existing glomerulosclerosis by inhibition of aldosterone. J. Am. Soc. Nephrol..

[7] Nath K.A., Kren S.M., Hostetter T.H. (1986). Dietary protein restriction in established renal injury in the rat. Selective role of glomerular capillary pressure in progressive glomerular dysfunction. J. Clin. Investig..

[8] Brenner B.M., Meyer T.W., Hostetter T.H. (1982). Dietary protein intake and the progressive nature of kidney disease: The role of hemodynamically mediated glomerular injury in the pathogenesis of progressive glomerular sclerosis in aging, renal ablation, and intrinsic renal disease. N. Engl. J. Med..

[9] Bellizzi V. (2013). Low-protein diet or nutritional therapy in chronic kidney disease?. Blood Purif..

[10] Kusano K., Segawa H., Ohnishi R., Fukushima N., Miyamoto K. (2008). Role of low protein and low phosphorus diet in the progression of chronic kidney disease in uremic rats. J. Nutr. Sci. Vitam..

[11] Neves K.R., Graciolli F.G., dos Reis L.M., Pasqualucci C.A., Moyses R.M., Jorgetti V. (2004). Adverse effects of hyperphosphatemia on myocardial hypertrophy, renal function, and bone in rats with renal failure. Kidney Int..

[12] Finch J.L., Lee D.H., Liapis H., Ritter C., Zhang S., Suarez E., Ferder L., Slatopolsky E. (2013). Phosphate restriction significantly reduces mortality in uremic rats with established vascular calcification. Kidney Int..

[13] Cupisti A., Bolasco P., D’Alessandro C., Giannese D., Sabatino A., Fiaccadori E. (2021). Protection of residual renal function and nutritional treatment: First step strategy for reduction of uremic toxins in end-stage kidney disease patients. Toxins.

[14] Yan B., Su X., Xu B., Qiao X., Wang L. (2018). Effect of diet protein restriction on progression of chronic kidney disease: A systematic review and meta-analysis. PLoS ONE.

[15] Klahr S., Levey A.S., Beck G.J., Caggiula A.W., Hunsicker L., Kusek J.W., Striker G. (1994). The effects of dietary protein restriction and blood-pressure control on the progression of chronic renal disease. Modification of diet in renal disease study group. N. Engl. J. Med..

[16] Hu M.C., Shi M., Cho H.J., Adams-Huet B., Paek J., Hill K., Shelton J., Amaral A.P., Faul C., Taniguchi M. (2015). Klotho and phosphate are modulators of pathologic uremic cardiac remodeling. J. Am. Soc. Nephrol..

[17] Hu M.C., Shi M., Cho H.J., Zhang J., Pavlenco A., Liu S., Sidhu S., Huang L.J., Moe O.W. (2013). The erythropoietin receptor is a downstream effector of Klotho-induced cytoprotection. Kidney Int..

[18] Shi M., Flores B., Gillings N., Bian A., Cho H.J., Yan S., Liu Y., Levine B., Moe O.W., Hu M.C. (2016). alphaKlotho mitigates progression of AKI to CKD through activation of autophagy. J. Am. Soc. Nephrol..

[19] Ding Y., Kim S., Lee S.Y., Koo J.K., Wang Z., Choi M.E. (2014). Autophagy regulates TGF-beta expression and suppresses kidney fibrosis induced by unilateral ureteral obstruction. J. Am. Soc. Nephrol..

[20] Moustakas A., Souchelnytskyi S., Heldin C.H. (2001). Smad regulation in TGF-beta signal transduction. J. Cell Sci..

[21] Di Marco G.S., Hausberg M., Hillebrand U., Rustemeyer P., Wittkowski W., Lang D., Pavenstadt H. (2008). Increased inorganic phosphate induces human endothelial cell apoptosis in vitro. Am. J. Physiol. Renal. Physiol..

[22] Meleti Z., Shapiro I.M., Adams C.S. (2000). Inorganic phosphate induces apoptosis of osteoblast-like cells in culture. Bone.

[23] Park J.W., Yook J.M., Ryu H.M., Choi S.Y., Morishita M., Do J.Y., Park S.H., Kim C.D., Choi J.Y., Chung H.Y. (2011). Phosphate-induced apoptosis in human peritoneal mesothelial cells in vitro. Am. J. Nephrol..

[24] Priante G., Gianesello L., Ceol M., Del Prete D., Anglani F. (2019). Cell death in the kidney. Int. J. Mol. Sci..

[25] Da J., Xie X., Wolf M., Disthabanchong S., Wang J., Zha Y., Lv J., Zhang L., Wang H. (2015). Serum phosphorus and progression of CKD and mortality: A meta-analysis of cohort studies. Am. J. Kidney Dis..

[26] Lee Y.J., Okuda Y., Sy J., Obi Y., Kang D.H., Nguyen S., Hsiung J.T., Park C., Rhee C.M., Kovesdy C.P. (2019). Association of mineral bone disorder with decline in residual kidney function in incident hemodialysis patients. J. Bone Miner. Res..

[27] Ibels L.S., Alfrey A.C., Haut L., Huffer W.E. (1978). Preservation of function in experimental renal disease by dietary restriction of phosphate. N. Engl. J. Med..

[28] Selamet U., Tighiouart H., Sarnak M.J., Beck G., Levey A.S., Block G., Ix J.H. (2016). Relationship of dietary phosphate intake with risk of end-stage renal disease and mortality in chronic kidney disease stages 3-5: The modification of diet in renal disease study. Kidney Int..

[29] Block G.A., Wheeler D.C., Persky M.S., Kestenbaum B., Ketteler M., Spiegel D.M., Allison M.A., Asplin J., Smits G., Hoofnagle A.N. (2012). Effects of phosphate binders in moderate CKD. J. Am. Soc. Nephrol..

[30] Dhillon-Jhattu S., Sprague S.M. (2018). Should phosphate management be limited to the KDIGO/ KDOQI guidelines?. Semin. Dial..

[31] Di Iorio B., Di Micco L., Torraca S., Sirico M.L., Russo L., Pota A., Mirenghi F., Russo D. (2012). Acute effects of very-low-protein diet on FGF23 levels: A randomized study. Clin. J. Am. Soc. Nephrol..

[32] Goto S., Nakai K., Kono K., Yonekura Y., Ito J., Fujii H., Nishi S. (2014). Dietary phosphorus restriction by a standard low-protein diet decreased serum fibroblast growth factor 23 levels in patients with early and advanced stage chronic kidney disease. Clin. Exp. Nephrol..

[33] Di Iorio B.R., Bellizzi V., Bellasi A., Torraca S., D’Arrigo G., Tripepi G., Zoccali C. (2013). Phosphate attenuates the anti-proteinuric effect of very low-protein diet in CKD patients. Nephrol. Dial. Transpl..

[34] Ix J.H., Anderson C.A., Smits G., Persky M.S., Block G.A. (2014). Effect of dietary phosphate intake on the circadian rhythm of serum phosphate concentrations in chronic kidney disease: A crossover study. Am. J. Clin. Nutr..

[35] Shen Z.J., Hu J., Shiizaki K., Kuro-o M., Malter J.S. (2016). Phosphate-induced renal fibrosis requires the prolyl isomerase Pin1. PLoS ONE.

[36] Doi S., Zou Y., Togao O., Pastor J.V., John G.B., Wang L., Shiizaki K., Gotschall R., Schiavi S., Yorioka N. (2011). Klotho inhibits transforming growth factor-beta1 (TGF-beta1) signaling and suppresses renal fibrosis and cancer metastasis in mice. J. Biol. Chem..

[37] Satoh M., Nagasu H., Morita Y., Yamaguchi T.P., Kanwar Y.S., Kashihara N. (2012). Klotho protects against mouse renal fibrosis by inhibiting Wnt signaling. Am. J. Physiol. Renal. Physiol..

[38] Zou D., Wu W., He Y., Ma S., Gao J. (2018). The role of klotho in chronic kidney disease. BMC Nephrol..

[39] Koh N., Fujimori T., Nishiguchi S., Tamori A., Shiomi S., Nakatani T., Sugimura K., Kishimoto T., Kinoshita S., Kuroki T. (2001). Severely reduced production of klotho in human chronic renal failure kidney. Biochem. Biophys. Res. Commun..

[40] Morishita K., Shirai A., Kubota M., Katakura Y., Nabeshima Y., Takeshige K., Kamiya T. (2001). The progression of aging in klotho mutant mice can be modified by dietary phosphorus and zinc. J. Nutr..

[41] Ramesh S., Wildey G.M., Howe P.H. (2009). Transforming growth factor beta (TGFbeta)-induced apoptosis: The rise & fall of Bim. Cell Cycle.

[42] August P., Suthanthiran M. (2003). Transforming growth factor beta and progression of renal disease. Kidney Int..

[43] Custodio M.R., Koike M.K., Neves K.R., dos Reis L.M., Graciolli F.G., Neves C.L., Batista D.G., Magalhaes A.O., Hawlitschek P., Oliveira I.B. (2012). Parathyroid hormone and phosphorus overload in uremia: Impact on cardiovascular system. Nephrol. Dial. Transpl..

[44] Qiu T., Wu X., Zhang F., Clemens T.L., Wan M., Cao X. (2010). TGF-beta type II receptor phosphorylates PTH receptor to integrate bone remodelling signalling. Nat. Cell Biol..

[45] Meng X.M., Chung A.C., Lan H.Y. (2013). Role TGF-Beta/BMP-7/Smad pathways in renal diseases. Clin. Sci..

[46] Chung A.C., Zhang H., Kong Y.Z., Tan J.J., Huang X.R., Kopp J.B., Lan H.Y. (2010). Advanced glycation end-products induce tubular CTGF via TGF-beta-independent Smad3 signaling. J. Am. Soc. Nephrol..

[47] Yang F., Chung A.C., Huang X.R., Lan H.Y. (2009). Angiotensin II induces connective tissue growth factor and collagen I expression via transforming growth factor-beta-dependent and -independent Smad pathways: The role of Smad3. Hypertension.

[48] Rojas E., Carlini R.G., Clesca P., Arminio A., Suniaga O., De Elguezabal K., Weisinger J.R., Hruska K.A., Bellorin-Font E. (2003). The pathogenesis of osteodystrophy after renal transplantation as detected by early alterations in bone remodeling. Kidney Int..

[49] Jilka R.L., Weinstein R.S., Bellido T., Roberson P., Parfitt A.M., Manolagas S.C. (1999). Increased bone formation by prevention of osteoblast apoptosis with parathyroid hormone. J. Clin. Investig..

[50] Kang R., Zeh H.J., Lotze M.T., Tang D. (2011). The Beclin 1 network regulates autophagy and apoptosis. Cell Death Differ..

[51] Choi A.M., Ryter S.W., Levine B. (2013). Autophagy in human health and disease. N. Engl. J. Med..

[52] Giampieri F., Afrin S., Forbes-Hernandez T.Y., Gasparrini M., Cianciosi D., Reboredo-Rodriguez P., Varela-Lopez A., Quiles J.L., Battino M. (2019). Autophagy in human health and disease: Novel therapeutic opportunities. Antioxid. Redox Signal..

[53] Lamb C.A., Yoshimori T., Tooze S.A. (2013). The autophagosome: Origins unknown, biogenesis complex. Nat. Rev. Mol. Cell Biol..

[54] Peng Y.F., Shi Y.H., Ding Z.B., Ke A.W., Gu C.Y., Hui B., Zhou J., Qiu S.J., Dai Z., Fan J. (2013). Autophagy inhibition suppresses pulmonary metastasis of HCC in mice via impairing anoikis resistance and colonization of HCC cells. Autophagy.

[55] Jia Y., Wu C., Kim J., Kim B., Lee S.J. (2016). Astaxanthin reduces hepatic lipid accumulations in high-fat-fed C57BL/6J mice via activation of peroxisome proliferator-activated receptor (PPAR) alpha and inhibition of PPAR gamma and Akt. J. Nutr. Biochem..

[56] Fujimura R., Yamamoto T., Takabatake Y., Takahashi A., Namba-Hamano T., Minami S., Sakai S., Matsuda J., Hesaka A., Yonishi H. (2020). Autophagy protects kidney from phosphate-induced mitochondrial injury. Biochem. Biophys. Res. Commun..

[57] Arias S.C., Valente C.P., Machado F.G., Fanelli C., Origassa C.S., de Brito T., Camara N.O., Malheiros D.M., Zatz R., Fujihara C.K. (2013). Regression of albuminuria and hypertension and arrest of severe renal injury by a losartan-hydrochlorothiazide association in a model of very advanced nephropathy. PLoS ONE.

[58] Teles F., Machado F.G., Ventura B.H., Malheiros D.M., Fujihara C.K., Silva L.F., Zatz R. (2009). Regression of glomerular injury by losartan in experimental diabetic nephropathy. Kidney Int..

[59] Jepsen F.L., Mortensen P.B. (1979). Interstitial fibrosis of the renal cortex in minimal change lesion and its correlation with renal function. A quantitative study. Virchows Arch. A Pathol. Anat. Histol..

